# Handgrip Strength Asymmetry and Weakness Are Differentially Associated with Functional Limitations in Older Americans

**DOI:** 10.3390/ijerph17093231

**Published:** 2020-05-06

**Authors:** Kyle Collins, Nathaniel Johnson, Lukus Klawitter, Roman Waldera, Sherri Stastny, William J. Kraemer, Bryan Christensen, Ryan McGrath

**Affiliations:** 1 Department of Health, Nutrition, and Exercise Sciences, North Dakota State University, Fargo, ND 58105, USA; kyle.s.collins@ndsu.edu (K.C.); nathaniel.johnson.4@ndsu.edu (N.J.); lukus.klawitter@ndsu.edu (L.K.); roman.w.waldera@ndsu.edu (R.W.); sherri.stastny@ndsu.edu (S.S.); bryan.christensen.1@ndsu.edu (B.C.); 2 Department of Human Sciences, The Ohio State University, Columbus, OH 43210, USA; kraemer.44@osu.edu

**Keywords:** aging, geriatrics, muscle strength, muscle strength dynamometer, nutrition surveys

## Abstract

*Background:* Handgrip strength (HGS) is a convent measure of strength capacity and associated with several age-related health conditions such as functional disability. Asymmetric strength between limbs has been linked to diminished function. Therefore, both HGS asymmetry and weakness could be associated with functional disability. We examined the associations of HGS asymmetry and weakness on functional limitations in a nationally representative sample of older Americans. *Methods*: Data were analyzed from 2689 adults ≥ 60 years who participated in the 2011–2012 and 2013–2014 waves of the National Health and Nutrition Examination Survey. Weakness was defined as HGS < 26 kg for men and < 16 kg for women. Asymmetry was determined from the ratio of the dominant and non-dominant HGS. Those with HGS ratio 0.9–1.1 were considered as having HGS symmetry, and those outside this range had asymmetry. *Results:* Compared to those with symmetric HGS and were not weak, those with weakness alone, and both weakness and HGS asymmetry had 2.47 (95% confidence interval [CI]: 1.14–5.35) and 3.93 (CI: 1.18–13.07) greater odds for functional limitations, respectively. However, HGS asymmetry alone was not associated with functional limitations (odds ratio: 0.80; CI: 0.62–1.03). *Conclusion:* The use of HGS asymmetry in protocols could improve the prognostic value of handgrip dynamometers.

## 1. Introduction

About 40% of older adults are currently living with a disability [1], and by the year 2050 it is estimated that 70 million Americans, about one fifth of the country’s population, will be aged over 65 years [2]. Moreover, those living with a functional limitation are at greater risk for additional losses in functioning [3]. If the prevalence of disability remains unchanged, in 2050, approximately 28 million Americans 65 years or older could be living with a disability [2], indicating the importance for early risk assessment and intervention. Maximal handgrip strength (HGS) serves as a convenient measure of neuromuscular integrity and function, making it a clinically-viable screening tool for several health conditions during aging [4].

Specifically, HGS is related to various functions of basic activities of daily living (BADL) [5,6] and cognitive function [7]. Previous research has found that instrumental activities of daily living (i.e., independent living tasks (IADLs)), demand high levels of cognitive function [8], and these findings suggest another pathway in which HGS may be related to different types of functional limitations. Although inabilities to perform IADLs and BADLs, and worsened physical and mental health, have been related to chronic health conditions in older adults [9], these same associations have not been made in relation to strength asymmetries.

Most research including HGS has examined either maximal or mean strength in one hand [10,11]; however, neuromuscular control during movement is proficient on the dominant side, and natural differences exist between hands depending on task complexity [12,13]. Regarding strength asymmetries, most research has focused on the lower body. Muscle activation asymmetry between dominant and non-dominant legs is linked to higher risk for falls [14] and sarcopenia [15]. Another study assessing power output and strength in younger adults, older non-fallers, and older fallers found that older fallers showed greater trends toward power asymmetry in the lower limbs and increased risk of sarcopenia [15]. Leg muscle fatigue asymmetry also increased demands on cognitive attention and ability [16], again showing a connection between asymmetry and nervous system function. Therefore, HGS asymmetry could be associated with a greater risk of functional disability.

The relationship between HGS asymmetry and functional limitations is supported by studies that have shown associations between physical decline [17], and loss of cognitive function [18] with disability, and by others who have determined that asymmetries in either lean body mass or nervous system function are related to worsened performance [17]. In support of this notion, in older Koreans, asymmetric distribution of lower-body lean mass is related to decreased gait speed [19], although lower-body strength asymmetries were not related to decreased mobility in older Americans [20].

A recent longitudinal investigation found that HGS asymmetry and weakness potentiated the risk for functional disability [21]. The findings from this study suggest that HGS asymmetry may factor into elevated functional disability risk. Given that HGS is differentially associated with limitations in individual functional tasks (e.g., grocery shopping, taking medications, preparing hot meals, etc.) [8], evaluating how HGS asymmetry is associated with other aspects of function may provide detailed insights into this association. Nonetheless, HGS asymmetry could be a more robust screening method for functional disability compared to using maximal values for a single hand. The purposes of this study were to determine the associations of HGS asymmetry and weakness on 1) functional limitations (i.e., aggregated) and 2) limitations in each aspect of function (i.e., BADL, IADL, leisure and social activities, lower extremity mobility, general physical tasks) in a nationally representative sample of older Americans. We hypothesize that those with HGS asymmetry and weakness will have greater odds for functional limitations and limitations in each aspect of function.

## 2. Materials and Methods

### 2.1. Participants

Publicly available data from 2899 adults aged at least 60 years with measures of HGS on both hands and who identified as either right- or left-handed were analyzed from the 2011–2012 and 2013–2014 waves of the National Health and Nutrition Examination Survey (NHANES). The NHANES is a program of studies designed to assess the health and nutrition status of Americans [22]. Mobile examination centers traveled to locations throughout the United States. Trained interviewers completed health interviews in the residences of participants using computer-aided interview systems and participants visited mobile examination centers for more detailed examinations [23].

Oversampling occurred for those aged at least 60 years, non-Hispanic Asians, non-Hispanic Blacks, and Hispanics to produce reliable data that better represented these demographics in the United States. Overall interview response rates were ≥68.5% for each wave of the NHANES included in our analyses [24]. The NHANES utilizes a complex, four-stage probability sampling design to generate a representative sample of non-institutionalized Americans. Sample weights were used in our analyses to account for the sampling methods and produce an unbiased national estimate [22,25]. Written informed consent was provided by participants, and NHANES protocols were approved by the National Center for Health Statistics Research Ethics Review Board (Protocol #2011-17).

### 2.2. Measures

#### 2.2.1. Variables of Functionality

Respondents told interviewers if they had no difficulty, some difficulty, much difficulty, or were unable to perform 19 tasks from five different aspects of function. These aspects included: (1) BADLs (“getting in and out of bed”, “using a fork, knife, and cup”, “walking between rooms on the same floor”, “dressing yourself”), (2) IADLs (“house chores”, “managing money”, “preparing meals”), (3) leisure and social activities (“going out to movies and events”, “leisure activities at home”, “attending social events”), (4) lower extremity mobility (“walking up 10 steps”, “walking for a quarter mile”), and (5) general physical tasks (“grasping or holding small objects”, “lifting or carrying”, “reaching up and overhead”, “sitting for long periods of time”, “standing for long periods of time”, “standing up from an armless chair”, “stooping, crouching, kneeling”).

Participants were considered as having a functional limitation if they reported having some difficulty, much difficulty, or were unable to perform any of the 19 tasks. Likewise, participants were considered as having a limitation in an aspect of function if they indicated having some difficulty, much difficulty, or were unable to perform any of the tasks listed within each aspect of function. Several investigations have used such criteria for defining functional limitations with NHANES data [26,27,28,29,30,31].

#### 2.2.2. Handgrip Strength Variables

A practice trial at sub-maximal effort was completed by participants to determine if the dynamometer was fitted to their hand size and to confirm understanding of the HGS test protocol. Interviewers that administered HGS tests were trained for protocols and calibration procedures. Participants reported their hand dominance and abilities to complete HGS protocols before testing, including if they had surgery to the hands or wrists within the previous three months that prohibited them from HGS testing. Interviewers instructed participants to stand with their feet hip width apart and hold the dynamometer away from their body with arm at side with palm facing leg so that the dynamometer did not contact the body (unless physically unable). Participants were encouraged to squeeze the handle of the dynamometer hard and quickly.

The decision to start testing on the dominant or non-dominant hand was randomized. Each person squeezed the dynamometer with maximal effort, exhaling while squeezing, and then released the skeletal musculature. An HGS measurement was completed three times on each hand, alternating between hands, with a minute of rest between measures on the same hand. A digital handgrip dynamometer (Takei Dynamometer Model T.K.K.5401; Akiha-Ku, Japan) was used to measure HGS. Additional details about the NHANES HGS protocols were previously published [32,33].

The single highest value regardless of hand dominance was used for determining weakness. Men with maximal HGS < 26 kg and women with maximal HGS < 16 kg were considered weak [34]. The highest recorded handgrip values from the dominant and non-dominant hands were used to calculate HGS ratio *(dominant HGS (kg) / non-dominant HGS (kg))*. Although HGS may differ between hands and depend on hand dominance [35], the “10% rule” suggests that the HGS of the dominant hand is about 10% stronger than the strength of the non-dominant hand [36]. Those who had HGS ratio <0.90 or >1.10 were considered as having asymmetric HGS, while those with HGS ratio 0.90–1.10 had HGS symmetry.

#### 2.2.3. Covariates

Age, sex, ethnicity (Hispanic, non-Hispanic Black, non-Hispanic White, other), and marital status (married, not-married) were self-reported. Standing height was measured with a fixed stadiometer, and body weight was collected with a digital scale (Mettler-Toledo International Inc.; Columbus, OH). Body mass index was calculated as body weight in kg divided by height in m^2^ (kg/m^2^). Those with a body mass index ≥ 30 kg/m^2^ were considered obese [37]. Respondents indicated if a healthcare provider had ever diagnosed them with diabetes, stroke, or arthritis. Moreover, respondents revealed if they experienced confusion or memory loss that was happening more often or getting worse. Participants were asked whether they had smoked at least 100 cigarettes in their lifetime (smoking history) and if they were currently smoking cigarettes. A single-item measure of self-perceived health was used wherein respondents rated their general health as either “excellent”, “very good”, “good”, “fair”, or “poor”. Depressive symptoms were assessed with the validated 9-item Patient Health Questionnaire. Each item was scored from 0–3 with higher scores indicating more severe depressive symptoms. The sum of scores across items was included in the analyses, and those with scores ≥ 10 were considered as having depression [38]. Those with missing covariates were excluded (*n* = 210).

#### 2.2.4. Statistical Analyses

All analyses were conducted with SAS 9.4 software (SAS Institute; Cary, NC, USA). Participants were categorized into handgrip asymmetry and weakness groups by their HGS measurements: (1) symmetric HGS and not weak, (2) weakness alone, (3) asymmetric HGS alone, and (4) both weak and asymmetric HGS. To determine the associations of the weakness and HGS asymmetry groups with functional limitations, separate logistic regression models were run. A logistic regression model analyzed the associations of each weakness and HGS asymmetry group on functional limitations using the not weak and symmetric HGS as the reference. Similarly, individual logit regression models also evaluated the associations of each weakness and HGS asymmetry group on limitations in each aspect of function (i.e., BADLs, IADLs, leisure and social activities, lower extremity mobility activities, general physical activities) using the not weak and symmetric HGS asymmetry group as the reference. Logistic regression models produce odds ratios, and logistic models were selected for our analyses because our study design was cross-sectional and outcome variables were binary. All logit models were adjusted for sex, age, ethnicity, self-rated health, diabetes diagnosis, stroke diagnosis, arthritis diagnosis, obesity, self-perceived memory impairment, depression, current smoking status, smoking history, and marital status. A p-value of < 0.05 was used to determine statistical significance for all analyses.

## 3. Results

There were 2689 (92.8%) of participants included, and their descriptive characteristics are presented in Table 1.

**Figure 1:**
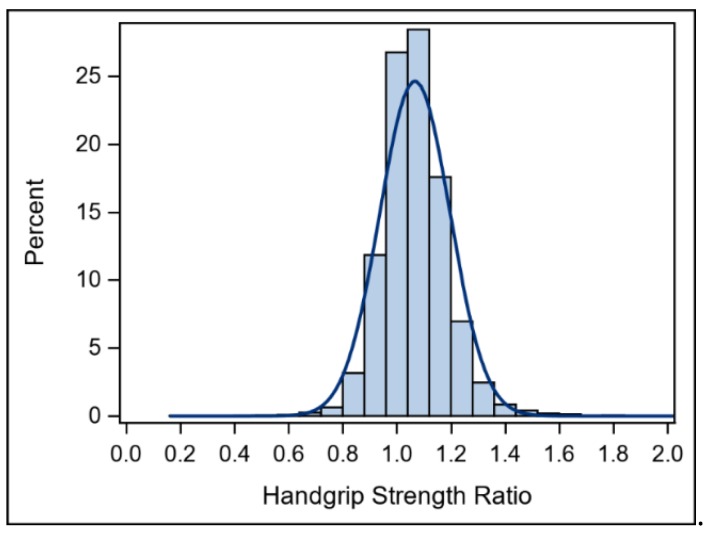
Histogram for handgrip strength ratio.

Appendix ATable A1 includes means and 95% confidence intervals for the descriptive characteristics to make comparisons across weakness and asymmetry groups. A histogram of HGS ratios is shown in Figure 1.

Those with HGS symmetry and were not weak (61.4%; 95% confidence interval (CI): 58.9%, 64.0%), and those who had HGS asymmetry only (62.0%; CI: 59.2%, 64.9%) had lower proportions of participants with functional limitations compared to those with weakness only (86.1%; CI: 78.1%, 94.1%) and both asymmetric HGS and weakness (92.2%; CI: 86.2%, 98.1%).

The results of the associations for HGS asymmetry and weakness on functional limitations are shown in Table 2.

Relative to those who were not weak and had HGS symmetry, those with weakness alone, and those with HGS asymmetry and weakness had differential associations for functional limitations. However, there were null findings for the association of those with HGS asymmetry alone and functional limitations.

Table 3 presents the results for the associations of the HGS asymmetry and weakness groups on limitations in each aspect of function. Those with weakness, and those with HGS asymmetry and weakness had differential associations for limitations in each aspect of function. There were no significant associations for those with HGS asymmetry alone and limitations in each aspect of function.

## 4. Discussion

The principal findings of this study were that weakness and HGS asymmetry were associated with greater odds for functional limitations in older Americans. Similar findings were observed when examining the associations between weakness and HGS asymmetry in each aspect of function; there were differential associations for those with both weakness and HGS asymmetry, and those with weakness alone on limitations in each aspect of functioning. Interestingly, those with only HGS asymmetry were not at increased odds for functional limitations. While natural strength asymmetries may exist between sides [14], the presence of both HGS asymmetry and weakness may exacerbate the odds for functional limitations. However, weakness could be driving these associations. Our findings suggest that including measures of HGS asymmetry in assessments of weakness may improve the prognostic utility of handgrip dynamometers for functional limitations.

In general, HGS has clinical value as it relates to entire body muscular strength [39], and weakness is associated with a number of important clinical health outcomes [40,41]. Due to these robust associations with clinical outcomes, HGS has been recommended as a routine vital sign for older adults [41]. However, findings of weakness and asymmetry may be more sensitive to the negative changes associated with aging than weakness alone. Measuring HGS weakness and asymmetry could also be added to other clinical screenings, such as measures of nutritional status, or physical function tests used to predict hospital stays [41]. The findings from this study indicate that handgrip asymmetry may improve a handgrip dynamometer’s ability for detecting functional declines. Similar to other elements of a comprehensive physical assessments, it would be beneficial to track weakness and asymmetry over time, giving that healthcare providers value information on patient physical trends.

In support of this notion, older Americans with both weakness and HGS asymmetry in our study were at increased odds for limitations in each aspect of functioning. Evidence suggests declines in HGS were associated with more dependence for completion of IADLs [8]. Neuropsychological and neuroanatomical changes such as decline in executive function or memory, hippocampal atrophy, and to a lesser extent white matter changes, may jeopardize one’s ability to perform IADLs [8]. The “common cause” hypothesis suggests that the same neural system deficits that could be responsible for limitations in IADLs may also be responsible for decreased muscle mass and HGS [42], which may help to explain our findings. Leisure and social activity, lower extremity mobility, and general physical activity task limitations were also more pronounced when individuals demonstrated asymmetry and weakness. Previous research is limited regarding older adults with weakness or asymmetry and the performance of physical tasks within those domains.

Indeed, the driving factors behind weakness and strength asymmetry are unclear. HGS asymmetries with weakness could be simply due to disuse of the non-dominant limb [43]. Alternatively, declines in grasping ability are reflective of reduced neural and motor system function [4,7]. For example, shifts in hand dominance and motor control could reflect changes in brain hemisphere functioning during aging [7]. When such shifts occur, muscle activation could also decline during a grip force task [44]. Therefore, difficulties in modulating the appropriate motor networks and decreasing motor performance exist in older adults [44]. Additionally, because the primary motor cortex in each hemisphere of the brain controls movement on the opposite side of the body, asymmetries between arms should be affected by asymmetries in the brain, and, in fact, asymmetries in somatosensory cortex have been reported [45].

Fortunately, various forms of exercise including strength and balance training, have been successful in mitigating some of the declines associated with aging [46,47,48]. While not the main outcome variable, a number of training studies have assessed HGS, with mixed results [46]. One study investigated the differences in high-speed versus low-speed resistance training in healthy older women and found that HGS improved [47]. Similarly, a 10-week study assessed the effects of aerobic and resistance exercise order in older men. Irrespective of exercise order, both groups saw increases in HGS and other measures of strength, walking ability, and flexibility [49]. Therefore, weakness could be reversible with exercise, but training just HGS may not address the root causation of asymmetry. In addition, a meta-analytical review found that a majority of training studies finding large improvements in HGS did so with multiple whole-body exercise modes such as strength, endurance, flexibility, and balance training [46].

The strength of this work includes the use of a nationally-representative sample and the statistical control of important covariates, yet we must acknowledge some of its limitations. Some self-report bias may have occurred within participant responses. For example, indication of hand dominance for each participant had to be established in order to calculate HGS ratio. In addition, levels of difficulty in the five aspects of function were self-reported by participants. However, self-reports are common in large epidemiological studies. NHANES data are cross-sectional, and protocols used for measuring HGS may be inconsistent with protocols used in similar investigations. Moreover, specific details in protocol manuals could be lacking. Longitudinal data for occupational history was unavailable. The null associations and wider confidence intervals could be attributed to lower sample sizes in the HGS asymmetry and weakness groups. Additionally, the “10% rule” was used to determine HGS asymmetry in participants; however, HGS ratios may vary between hands at an individual level [34]. Different cut-off points would likely affect the strength of the relationship between HGS asymmetry and weakness for functional limitations. More research is needed to determine if asymmetry is associated with function beyond weakness, and to decipher the difference between loss of muscle strength or sensorimotor ability. Further development of intervention strategies to combat functional decline and loss of HGS in older adults is merited.

## 5. Conclusions

Older adults who have both weakness and asymmetric HGS have greater odds for functional limitations. Similarly, those who were both weak had asymmetric HGS had greater odds for limitations in each aspect of function. Weakness could be driving these associations. Including HGS asymmetry in HGS protocols may help to improve the prognostic value of handgrip dynamometers and operationalization of strength capacity. Measures of HGS asymmetry will also not compromise the feasibility of HGS assessments because hand dominance and multiple measures of HGS are recorded in most HGS protocols.

## Figures and Tables

**Table 1:** Descriptive characteristics of the participants.

Variables	Overall (*n* = 2689)	Symmetric HGS and Not Weak (*n* = 1427)	Weak Only (*n* = 72)	Asymmetric HGS Only (*n* = 1113)	Asymmetric HGS and Weak (*n* = 77)
HGS (kg)	31.7 ± 10.0	32.7 ± 9.6	18.4 ± 5.3	32.4 ± 9.7	17.2 ± 5.2
HGS Ratio	1.06 ± 0.15	1.01 ± 0.05	1.01 ± 0.05	1.14 ± 0.20	1.11 ± 0.24
Age (years)	69.6 ± 6.8	68.9 ± 6.7	75.5 ± 6.1	69.5 ± 6.7	75.8 ± 5.9
Male (n (%))	1299 (48.2)	711 (49.8)	44 (61.1)	510 (45.8)	34 (44.1)
Ethnicity (n (%))					
Hispanic	490 (18.2)	251 (17.6)	10 (13.9)	215 (19.3)	14 (18.2)
Non-Hispanic Black	638 (23.7)	339 (23.7)	16 (22.2)	274 (24.6)	9 (11.7)
Non-Hispanic White	1296 (48.2)	700 (49.1)	34 (47.2)	517 (46.5)	45 (58.4)
Other	265 (9.9)	137 (9.6)	12 (16.7)	107 (9.6)	9 (11.7)
Obese (n (%))	1017 (37.8)	523 (36.6)	31 (43.0)	422 (40.1)	22 (28.5)
Diabetes (n (%))	625 (23.2)	324 (22.7)	26 (36.1)	252 (22.6)	23 (29.8)
Arthritis (n (%))	1319 (49.0)	662 (46.3)	33 (45.8)	575 (51.6)	49 (63.6)
Stroke (n (%))	186 (6.9)	78 (5.4)	15 (20.8)	82 (7.3)	11 (14.2)
Self-Perceived Memory Impairment (n (%))	382 (14.2)	188 (13.2)	19 (26.3)	153 (13.7)	22 (28.5)
Current Smoker (n (%))	334 (12.4)	183 (12.8)	8 (11.1)	133 (11.9)	10 (12.9)
Previous Smoker (n (%))	1022 (37.9)	539 (37.7)	29 (40.2)	426 (38.2)	28 (36.3)
Currently Married (n (%))	1474 (54.8)	812 (56.9)	33 (45.8)	601 (54.0)	28 (36.3)
Depressed (n (%))	227 (8.4)	107 (7.5)	10 (13.8)	102 (9.1)	8 (10.3)
Self-Rated Health (n (%))					
Excellent	208 (7.7)	118 (8.3)	4 (5.7)	80 (7.2)	6 (7.8)
Very Good	664 (24.7)	355 (24.9)	11 (15.2)	279 (25.1)	19 (24.7)
Good	1064 (39.5)	574 (40.2)	27 (37.5)	443 (39.8)	20 (26.0)
Fair	630 (23.5)	328 (23.0)	23 (31.9)	254 (22.8)	25 (32.4)
Poor	123 (4.6)	52 (3.6)	7 (9.7)	57 (5.1)	7 (9.1)
Functional Limitation (n (%))	1701 (63.2)	877 (61.4)	62 (86.1)	691 (62.0)	71 (92.2)

Note: HGS = handgrip strength.

**Table 2:** Association for the handgrip strength asymmetry and weakness groups on functional limitations.

Groups	Odds Ratio	95% Confidence Interval
Weakness Alone ^†^	2.47	1.14, 5.35
Handgrip Strength Asymmetry Alone ^†^	0.80	0.62, 1.03
Weak and Handgrip Strength Asymmetry ^†^	3.93	1.18, 13.07

^†^ Reference: Symmetric handgrip strength and not weak. Note: the model was adjusted for sex, age, ethnicity, self-rated health, diabetes diagnosis, stroke diagnosis, arthritis diagnosis, obesity, self-perceived memory impairment, depression, current smoking status, smoking history, and marital status.

**Table 3:** Associations for the handgrip strength asymmetry and weakness groups on limitations in each aspect of function.

Groups	Odds Ratio	95% Confidence Interval
*Activities of Daily Living* ^†^		
Weakness Only	3.80	1.80, 8.05
HGS Asymmetry Only	1.19	0.88, 1.61
HGS Asymmetry and Weakness	5.28	2.20, 12.70
*Instrumental Activities of Daily Living* ^†^		
Weakness Only	2.15	1.14, 4.06
HGS Asymmetry Only	1.01	0.77, 1.32
HGS Asymmetry and Weakness	4.24	1.78, 10.14
*Leisure and Social Activities* ^†^		
Weakness Only	2.57	1.26, 5.24
HGS Asymmetry Only	0.80	0.62, 1.03
HGS Asymmetry and Weakness	3.50	1.26, 9.74
*Lower Extremity Mobility Activities* ^†^		
Weakness Only	2.44	1.28, 4.67
HGS Asymmetry Only	0.79	0.59, 1.07
HGS Asymmetry and Weakness	2.85	1.13, 7.19
*General Physical Activities* ^†^		
Weakness Only	4.25	2.03, 8.90
HGS Asymmetry Only	0.96	0.74, 1.25
HGS Asymmetry and Weakness	3.55	1.45, 8.68

^†^ Reference: Symmetric handgrip strength and not weak. Note: the model was adjusted for sex, age, ethnicity, self-rated health, diabetes diagnosis, stroke diagnosis, arthritis diagnosis, obesity, self-perceived memory impairment, depression, current smoking status, smoking history, and marital status.

## Appendix A

**Table A1:** Descriptive characteristics of the participants.

Variables	Overall	Group 1	Group 2	Group 3	Group 4
HGS (kg)	31.7 (31.4, 32.1)	32.7 (32.2, 33.2)	18.4 (16.9, 19.4)	32.4 (31.8, 33.0)	17.2 (16.2, 18.6)
HGS Ratio	1.06 (1.06, 1.07)	1.01 (1.01, 1.02)	1.01 (1.00, 1.02)	1.14 (1.12, 1.15)	1.11 (1.05, 1.16)
Age (years)	69.6 (69.3, 69.8)	68.9 (68.6, 69.3)	75.5 (74.1, 77.0)	69.5 (69.1, 69.9)	75.8 (74.5, 77.2)
Male (n (%))	48.2 (46.4, 50.1)	49.8 (47.2, 52.3)	61.1 (49.8, 72.3)	45.8 (42.8, 48.7)	44.1 (33.0, 55.2)
Ethnicity (n (%))					
Hispanic	18.2 (16.7, 19.7)	17.6 (15.6, 19.6)	13.9 (5.9, 21.8)	19.3 (17.0, 21.6)	18.2 (9.5, 26.8)
Non-Hispanic Black	23.7 (22.1, 25.3)	23.7 (21.5, 25.9)	22.2 (12.6, 31.8)	24.6 (22.0, 27.1)	11.7 (4.5, 18.8)
Non-Hispanic White	48.2 (46.2, 50.0)	49.1 (46.4, 51.6)	47.2 (35.6, 58.7)	46.5 (43.5, 49.3)	58.4 (47.4, 69.4)
Other	9.9 (8.7, 10.9)	9.6 (8.0, 11.1)	16.7 (8.0, 25.2)	9.6 (7.8, 11.3)	11.7 (4.5, 18.8)
Obese (n (%))	37.8 (35.9, 39.6)	36.6 (34.1, 39.1)	43.0 (31.6, 54.4)	40.1 (36.7, 42.5)	28.5 (18.4, 38.6)
Diabetes (n (%))	23.2 (21.6, 24.8)	22.7 (20.5, 24.8)	36.1 (25.0, 47.2)	22.6 (20.1, 25.1)	29.8 (19.6, 40.0)
Arthritis (n (%))	49.0 (47.1, 50.9)	46.3 (43.7, 48.9)	45.8 (34.3, 57.3)	51.6 (48.7, 54.6)	63.6 52.8, 74.3)
Stroke (n (%))	6.9 (5.9, 7.8)	5.4 (4.2, 6.6)	20.8 (11.4, 30.2)	7.3 (5.8, 8.9)	14.2 (6.4, 22.1)
Self-Perceived Memory Impairment (n (%))	14.2 (12.9, 15.5)	13.2 (11.4, 14.9)	26.3 (16.2, 36.5)	13.7 (11.7, 15.7)	28.5 (18.4, 38.6)
Current Smoker (n (%))	12.4 (11.1, 13.6)	12.8 (11.0, 14.5)	11.1 (3.8, 18.3)	11.9 (10.0, 13.8)	12.9 (5.4, 20.5)
Previous Smoker (n (%))	37.9 (36.1, 39.8)	37.7 (35.2, 40.2)	40.2 (28.9, 51.6)	38.2 (35.4, 41.1)	36.3 (25.6, 47.1)
Currently Married (n (%))	54.8 (52.9, 56.6)	56.9 (54.2, 59.4)	45.8 (34.3, 57.3)	54.0 (51.0, 56.9)	36.3 (25.6, 47.1)
Depressed (n (%))	8.4 (7.4, 9.5)	7.5 (6.1, 8.9)	13.8 (5.9, 21.8)	9.1 (7.4, 10.8)	10.3 (3.5, 17.2)
Self-Rated Health (n (%))					
Excellent	7.7 (6.7, 8.7)	8.3 (6.8, 9.6)	5.7 (0.2, 10.8)	7.2 (5.6, 8.7)	7.8 (1.8, 13.7)
Very Good	24.7 (23.0, 26.3)	24.9 (22.6, 27.1)	15.2 (6.9, 23.5)	25.1 (22.5, 27.6)	24.7 (15.0, 34.3)
Good	39.5 (37.7, 41.4)	40.2 (37.6, 42.7)	37.5 (26.3, 48.6)	39.8 (36.9, 42.6)	26.0 (16.1, 35.7)
Fair	23.5 (21.8, 25.0)	23.0 (20.8, 25.2)	31.9 (21.1, 42.7)	22.8 (20.3, 25.2)	32.4 (22.0, 42.9)
Poor	4.6 (3.7, 5.3)	3.6 (2.6, 4.6)	9.7 (2.8, 16.5)	5.1 (3.8, 6.4)	9.1 (2.6, 15.5)
Functional Limitation (n (%))	63.2 (61.4, 65.0)	61.4 (58.9, 64.0)	86.1 (78.1, 94.1)	62.0 (59.2, 64.9)	92.2 (86.2, 98.1)

Note: Group 1 = symmetric HGS and not weak; Group 2 = weakness alone; Group 3 = HGS asymmetry alone; Group 4 = HGS asymmetry and weakness; HGS = handgrip strength.
